# Supplementary material for: Development of a simple method for differential delivery of volatile anesthetics to the spinal cord of the rabbit
Source: PLoS One. 2020 Feb 24;15(2):e0223700. doi: 10.1371/journal.pone.0223700 (PMC7039460; doi:10.1371/journal.pone.0223700)

Manuscript Click here to download Manuscript Manuscript-Development.docx

Development of a simple method for differential delivery of volatile anaesthetics to the spinal cord of the rabbit

Peng Zhang, Yao Li, Ting Xu*

Department of Anesthesiology, Sichuan Academy of Medical Sciences & Sichuan Provincial People's Hospital, Chengdu, Sichuan Province, China

*Correspondence: Ting Xu

Email: xt1979@gmail.com

Funding: This project was supported by the Young Talents Foundation of Sichuan Provincial People's Hospital (2017QN10)

Abstract

Emulsified volatile anaesthetics can be easily injected into the circulation and eliminated from blood ~~to~~ in the lungs. Taking advantage of this unique pharmacokinetics, we aimed to develop a less ~~trauma~~ traumatic model for preferential delivery of volatile anaesthetics to the spinal cord with an intact CNS and circulatory system in the rabbit. Sixteen New Zealand White rabbits were randomly assigned to the isoflurane and sevoflurane group. A catheter for delivering emulsified isoflurane (8mg/kg/h) or sevoflurane (12mg/kg/h) to the spinal cord was placed into the descending aorta in all rabbits. The concentration and partial pressure of isoflurane and sevoflurane in the jugular and femoral vein were measured. Our results showed that the partial pressure of isoflurane was 3.91±1.11 mmHg in the jugular vein and 12.61±1.60 mmHg (1.0MAC) in the femoral vein. The partial pressure of sevoflurane was 3.89±1.00 mmHg in the jugular vein and 19.92±1.84mmHg (1.0MAC) in the femoral vein. There was significant difference ~~regarding~~ between ~~the~~ jugular and femoral vein partial pressure ~~of~~ for both isoflurane and sevoflurane ~~between jugular and femoral veins in two~~ groups (both $P<0.001$). In conclusion, a simple method has been successfully developed to selectively deliver isoflurane and sevoflurane to the spinal cord in the rabbit. Before acting on the brain, 69% isoflurane and 81% sevoflurane were removed from body via lungs. This method can be used to investigate ~~the pharmacological properties~~ sites and mechanisms of volatile anesthetic~~s~~ action.

Abstract

Induction

Volatile anaesthetics can induce a variety of reversible, clinically important effects such as amnesia, hypnosis and immobility[1, 2]. To determine if an effect is due to brain, spinal cord, peripheral nerves or both [but this model cannot distinguish between spinal cord and peripheral nerve effects], we need a differential anaesthetic delivery model[3, 4]. To solve this problem, previous research~~es~~ ~~have~~ has ~~introduced~~ utilized several selectively anaesthetized models in the goat, dog and rabbit, using cardiopulmonary bypass technology [5-7] [the goat model did not show evidence that trauma affected the results, MAC was in the normal range and did not change before or after bypass. The main advantage to the present rabbit model is that it is less labor intensive intensive and costly] ~~But, these models limited by the severe trauma which could destroy the physiological structure of the cerebral and spinal cord circulation of experimental animals.~~ However, our model permits selective perfusion of the brain versus spinal cord that is less costly and labor intensive while obviating most of the trauma associated with prior models.

Rabbits have a unique spinal cord circulation in that each spinal cord segment is supplied by a single corresponding radicular artery arising from the aorta[8, 9].In addition, the blood supply to the thoracolumbar region (below T_3) of spinal cord originates from the thoracic and abdominal aorta in the rabbit [10-12]. ~~Emulsified~~ volatile anaesthetics can be ~~is that liquid anaesthetic dissolves~~ dissolved in an ~~emulsion~~ emulsifiying agent, which ~~and~~ produces anaesthesia as effectively as a vaporized and inhaled~~liquid~~ one [13, 14]. ~~What's important is that, e~~ Emulsified volatile anaesthetic can be directly injected into the circulation ~~as same as~~similar to intravenous drugs, but ~~and~~ eliminated via the lungs[15, 16]. Based on these findings, we aimed to

Induction

establish a ~~less trauma~~ simpler model for differential delivery of volatile anaesthetics (isoflurane and sevoflurane) to the spinal cord with an intact CNS [prior models have an intact CNS] and circulatory system in the rabbit.

Materials and methods

Emulsified isoflurane and sevoflurane preparation

Isoflurane and sevoflurane emulsion were prepared according to a previously described formula[17, 18]. In brief, 1.6mL of liquid isoflurane or sevoflurane (Abbott, Shanghai, China) and 18.4mL of 30% intralipid (Baxter, Suzhou, China) were injected into a sealed sterile vial. Then the vial was violently shaken for 10min by a vibrator to solubilizse isoflurane or sevoflurane into the intralipid. The 8% emulsified isoflurane and sevoflurane were stored in 4°C refrigerator before use.

Animal preparation and surgical procedures

The study was approved by the Institutional Animal Care and Use Committee of Sichuan Provincial People's Hospital. Sixteen New Zealand White rabbits (male and female) weighting 2.0-3.0kg were randomly assigned to the isoflurane and sevoflurane group [8 rabbits per group?]. After intravenous injection of 30 mg/kg pentobarbital sodium into the left marginal ear vein, a ID 3.0# cuffed endotracheal tube was inserted. All rabbits were mechanical ventilated with 95% O_2 using an animal ventilator (Chengdu Techman Software Co.Ltd, Chengdu, China), with the tidal volume 8ml/kg, 40 breaths/min, to maintain the arterial carbon dioxide pressure (PaCO2) between 35 to 45 mmHg. A 22-gauge IV catheter (BD company, Sandy Utach, USA) was individually inserted into the left central ear and femoral artery to monitor the blood pressure in the upper and lower torso of the rabbit in each group.

An epidural catheter (TuoRen Medical Instrument Co., Ltd, Xinxiang, China) ~~which~~ was used to deliver emulsified isoflurane or sevoflurane to the spinal cord, and was placed into the descending aorta from the right femoral artery (Fig 1). The change of resistance was monitored during the catheter passing cranially along the aorta. The catheter would be withdrawn 0.5-1.0 cm, when the increased resistance was recorded. The placement of catheter tip was confirmed by autopsy after the experiment. In our study, the rabbit would be excluded from data analysis if the tip was located in the aortic arch level. The length of catheter between catheter tip and inguinal fold was measured.

The infusion rate was 8mg/kg/h in the isoflurane group and 12mg/kg/h in the sevoflurane group by a microinfusion pump. When the end-tidal isoflurane concentration (E_TISO) or end-tidal sevoflurane concentration (E_TSEVO) was stable and maintained for 20min, emulsified volatile anaesthetic was considered to reach steady state condition[15]. There were two methods to avoid re-breaching of isoflurane or sevoflurane into the brain: (a.) a higher inspiratory flow 2L/min (normal minute ventilation (MV) of rabbit is about 1L/min) (b.) an anaesthetic absorber made of activated charcoal in the inspiratory limb of the circuit.

Lactated Ringer's solution (6ml/kg/h) was administered through the left marginal ear vein in all rabbits. ~~Rectum~~ Rectal temperature was monitored and maintained at 37.0±1.0°C. During the experiment, heart rate (HR), mean arterial pressure (MAP),

electrocardiograph (ECG) and pulse oxygen saturation (SpO_2) were measured with

An epidural catheter (TuoRen Medical Instrument Co., Ltd, Xinxiang, China)

the BL-420E+ Data Acquisition and Analysis System (Chengdu Techman Software Co.Ltd, Chengdu, China). The E_TISO, E_TSEVO and E_TCO_2 (end-tidal CO_2) were monitored with an anesthetic gas analyzer M1026B (Philips Medizin Systeme Boblingen GmbH, Boblingen, Germany).

Collection of blood samples and gas chromatograph

Once the end-tidal concentration of isoflurane or sevoflurane was stable and maintained for 20min, 5-6ml blood was rapidly withdrawn from the jugular and femoral vein to determine the isoflurane or sevoflurane blood concentration (C). Samples were analyzed ~~in the~~ ~~blood,~~ using a gas chromatograph Agilent 4890D (Tegent Technology Ltd., Hong Kong, China) with ~~the~~ two-stage headspace equilibration methods[15]. The partial pressure of isoflurane and sevoflurane (P) in the different blood samples were calculated by the equation of $P= (C/\lambda_{b/g}\times760$ mmHg) ($\lambda_{b/g}$ =blood/gas partition coefficient). The C and P of all samples ~~was~~ were measured by a technician who was blinded to the samples. In our previous study, the partial pressure of 1.0 MAC (minimum alveolar concentration) isoflurane was 11.66±1.10 mmHg and the partial pressure of 1.0 MAC sevoflurane was 18.86±1.12 mmHg.

Statistical analysis

Statistical analyses were performed using SPSS 22.0. Each continuous variable was analyzed for its normal distribution and reported as mean ± SD. Repeated

measures ANOVA was used to evaluate MAP and HR among different time points in two groups. Pearson correlation coefficient was used to measure the linear relationship between $\lambda_{b/g}$ and the volume (mL) of the emulsified isoflurane or sevoflurane consumed. Student's t test and χ^2 test were also measured in our study [in which cases/for what was each of these two test used?]. A *P* value of less than 0.05 was considered statistically significant. A Bonferroni correction was made when necessary to correct for multiple testing.

Results

~~Sixteen rabbits finished the experiment~~Full data sets were collected from sixteen rabbits.~~ and all data were analyzed~~. The mean weight~~s~~ of the rabbits ~~were~~ was 2.38±0.26 kg in the isoflurane group and 2.46±0.24 kg in the sevoflurane group, which were not significantly different. ~~No significant difference was found between two groups~~ (P=0.532). The mean lengths of the inserted catheter (between catheter tip and inguinal fold) were 25.13±1.07 cm in the isoflurane group and 25.36±0.86 cm in the sevoflurane group. In the isoflurane group, two catheters (25%) were located at T_2 and six (75%) at T_3. In the sevoflurane group, four catheters (50%) were placed at T_2 and four (50%) at T_3 (Table 1). There was no significant difference with regard to the length of the catheter (P=0.608) and position of the catheter tips (P=0.631) between two groups.

No significant difference was found regarding MAP of the central ear artery among three different time points in two groups (P=0.169 for isoflurane group; P=0.390 for sevoflurane group). But MAP of the femoral artery at the midpoint of ~~emulsified anaesthetics~~ the infusion was significantly reduced compared with the other two time points (after induction and end of the study) in two groups (both P<0.001). The trend of MAP change between central ear artery and femoral artery was significantly different during the whole experiment in ~~two~~ both groups (~~both~~ P<0.001). However, there was no significant difference in the MAP between central ear artery and femoral artery during the whole experiment in two groups (P=0.062 for isoflurane group;

P=0.169 for sevoflurane group) (Table 2). No significant difference was found with

Results

regard to HR among three time points in the isoflurane group (*P*=0.091) and sevoflurane group (*P*=0.076) (Table 3).

The partial pressure of isoflurane was 3.91±1.11 mmHg in the jugular vein and 12.61±1.60 mmHg (1.0MAC) in the femoral vein. The partial pressure of sevoflurane was 3.89±1.00 mmHg in the jugular vein and 19.92±1.84mmHg (1.0MAC) in the femoral vein. ~~There was significant difference regarding t~~The partial pressure of ~~isoflurane and sevoflurane~~ between jugular and femoral vein ~~in two groups~~was significant for both the isoflurane and sevoflurane groups (both *P*<0.001). The ratio of partial pressure of isoflurane and sevoflurane between jugular vein and femoral vein ~~was showed~~ is shown in the Table 4.

There was a significant positive correlation between $\lambda_{b/g}$ and volume of the emulsified anaesthetics delivered (r=0.935, *P*<0.001 for isoflurane group; r=0.919, *P*=0.001 for sevoflurane group). The Linear regression equation was y=0.0869x+0.9688 (R^2=0.8723) in the isoflurane group (Fig 2) and y=0.0526x+0.3889 (R^2=0.8452) in the sevoflurane group (Fig 3) (y=volume and x= $\lambda_{b/g}$).

Discussion

The rabbit, with its medium size and homosegmental blood supply to the spinal cord, ~~make it~~ is an ideal animal for selectively anaesthetized models [10-12]. ~~Due to~~With the catheter inserted into the descending aorta, isoflurane and sevoflurane emulsion were able to be preferentially delivered to the thoracolumbar (below T_4) region of the spinal cord. ~~But b~~Before acting on the brain, the majority of ~~it were prior~~anesthetic was eliminated via the lungs. ~~According to our results, the rabbit model could exhale~~Using this model, 69% of isoflurane, or 81% of sevoflurane ~~by lungs, before delivering to~~was eliminated by the lungs before reaching the brain. Therefore, the ~~lower~~ torso Circulation below the T3 level ~~of the rabbit was successfully bypassed from~~was selectively perfused with anesthetic compared to the ~~upper torso part of the body~~ upper spinal cord and brain in our study.

When volatile anaesthetic reached a steady state conditions in the body, the partial pressure in the alveolar, blood and central nervous system is presumed to be in equilibrium with ~~each parts~~one an-other [15, 19]. In our study, to be specific, the partial pressure in the jugular and femoral veins ~~can reflect~~ reflect that in the brain and spinal cord, tissue r~~R~~espectively, under a steady state condition. Our results showed that the isoflurane partial pressure in the femoral vein was about 3.5-fold more than that in the jugular vein, and the ratio of sevoflurane was 5.3-fold. In other words, the partial pressure of isoflurane and sevoflurane in the spinal cord were 3.5-fold and 5.3-fold more than that in the brain tissue. Thus, our model could differentially deliver emulsified isoflurane

and sevoflurane to the spinal cord in the rabbit.

In *Yang*'s goat model [ref?], using similar technologies, emulsified isoflurane was selectively delivered to the spinal cord, with an approximate 46% reduction in the

isoflurane partial pressure in the brain [18]. ~~However, the partial pressure of~~ In our present study, brain concentrations of isoflurane ~~in the brain was only~~and sevoflurane were only ~~about~~ 31% ~~of that in the spinal cord, while the ratio of~~ and 19% of the torso (spinal cord) concentrations, respectively ~~sevoflurane was 19% in our study~~. Therefore Our rabbit model ~~seemed more~~was more ~~effective in~~ ~~removing anaesthetics~~selective compared to the ~~than~~ previous goat model using emulsified volatile anesthetics. There were several possible reasons: (a.) The oxygen consumption per kilogram of rabbit is ~~quite~~ larger than that of goats, and MV/kg of rabbit is about 2 times than that of goat [20]. It has established that increasing MV might contribute to remove volatile anaesthetics from blood to lungs [21]. (b.)Two methods mentioned (higher inspiratory flow and anaesthetic absorber) were used to avoid re-breaching of isoflurane or sevoflurane (c.) In our study, sevoflurane was ~~easier to be~~ more rapidly eliminated from body than isoflurane, because of lower blood gas solubility ($\lambda_{b/g}$ of sevoflurane=0.069; $\lambda_{b/g}$ of isoflurane=1.37) [22].

It is well known that $\lambda_{b/g}$ of 30% intralipid is remarkably larger than that of isoflurane or sevoflurane. So $\lambda_{b/g}$ of isoflurane and sevoflurane would significantly increase with infusion of lipid emulsion in our study. Our results indicated that 1 ml of 8% emulsified isoflurane increased $\lambda_{b/g}$ by 0.0869 at 37°C, and 0.0526 for sevoflurane $\lambda_{b/g}$. Some discrepancy ~~researches~~ in the literature showed that 1 ml of 8% emulsified isoflurane raised $\lambda_{b/g}$ by 0.0176 in the goat [18] and 0.0139 in the dog[15]. Obviously, the blood volume of rabbits is significantly less than that of goats or dogs[21]. Based on “volume fraction partition coefficient” theory, when 1ml of lipid emulsion was

administrated to those three animals, the change of rabbit $\lambda_{b/g}$ would be probably the [this sentence is unclear please clarify]

isoflurane partial pressure in the brain [18]. ~~However, the partial pressure of~~

biggest one [23]. But there was no similar report published regarding $\lambda_{b/g}$ of sevoflurane.

In our study, because of blood supply limitations to the spinal cored, only the thoracolumbar region (below T_3), not the entire spinal cord, could be selectively anaesthetized during aortic delivery of emulsified isoflurane and sevoflurane. ThoughAlthough the rabbit brain could not completely be separated selectively perfused from the entire spinal cord, our research still had few several advantages. Firstly, because of less trauma, compared with cardiopulmonary bypass, the integrity of circulation and CNS (central nervous system) can remain intact in our study[6, 7, 24]. Secondly, rabbits are common experimental animals and may have financial benefit. At the end of the study, all rabbits awoke and no fatalities was foundoccurred. This confirmed that infusion of emulsified isoflurane or sevoflurane produced a safe and reversible anaesthetic effect with no apparent pathological damage to the CNS and circulatory system [again, there was no evidence that CNS was compromised in the goat studies as recovery to normal MAC occurred following bypass].

In conclusion, a simple method has been successfully established that permitsto differential delivery of isoflurane and sevoflurane to the spinal cord with an intact CNS and circulatory system in rabbits. This model permitted was found to eliminate 69% isoflurane and 81% sevoflurane from blood to lungs, before acting on the brain. This allows us to determine if an anaesthesia action is due to brain or spinal cord effects. In addition,

rely on rabbit unique homosegmental blood supply to the spinal cord, this model can also be used to preferentially delivery of anaesthetics to the lumbar or sacro-coccygeal

biggest one [23]. But there was no similar report published regarding $\lambda_{b/g}$ of

[but it is already known VA act primarily in the spinal cord to exert immobility, so how would this add to our body of knowledge?]

[limitations of the model should be discussed, e.g., one cannot regulate anesthetic concentration to the brain, unlike the bypass models. This poses a n animal welfare concern as animals will likely become conscious when peri-Mac concentrations are delivered to the spinal cord, especially in rabbits that have higher anesthetic requirements compared to other mammals]

~~spinal cord, thereby permitting to investigate the effects of different each spinal cord~~ spinal cord, thereby permitting investigation of the effects of anesthetics on different spinal segments. ~~segment on anaesthesia action~~.

Author Contributions

Animal experiment: Peng Zhang and Yao Li

Data analysis: Yao Li

Writing-original draft: Peng Zhang

Writing-review and editing: Ting Xu

References

1. Campagna JA, Miller KW, Forman SA. Mechanisms of actions of inhaled anesthetics. N Engl J Med. 2003;348:2110-2124. https://doi.org/10.1056/NEJMra021261 PMID: 12761368
2. Antognini JF, Carstens EE. Anesthesia, amnesia, and the amygdala: reducing the fear of intraoperative awareness. Anesthesiology. 2005;102:711-712. https://doi.org/10.1097/00000542-200504000-00002 PMID: 15791096
3. Antognini JF, Schwartz K. Exaggerated anesthetic requirements in the preferentially anesthetized brain. Anesthesiology. 1993;79:1244-1249. https://doi.org/00000542-199312000-00015 PMID: 8267200
4. Yang J, Chai YF, Gong CY, Li GH, Luo N, Luo NF, et al. Further proof that the spinal cord, and not the brain, mediates the immobility produced by inhaled anesthetics. Anesthesiology. 2009;110:591-595. https://doi.org/10.1097/ALN.0b013e3181974bfd PMID: 19212265
5. Antognini JF, Jinks S, Buzin V, Carstens E. A method for differential delivery of intravenous drugs to the head and torso of the goat. Anesth Analg. 1998;87:1450-1452. https://doi.org/10.1097/00000539-199812000-00050 PMID: 9842849
6. Antognini JF, Kien ND. A method for preferential delivery of volatile anesthetics to the in situ goat brain. Anesthesiology. 1994;80:1148-1154. https://doi.org/10.1097/00000542-199405000-00024 PMID: 8017652

7. Atherley R, Weatherford V, Antognini JF, Jinks SL, Carstens E. A model for differential volatile anesthetic delivery to the upper and lower torso of the rabbit. J Pharmacol Toxicol Methods. 2004;50:145-152. https://doi.org/10.1016/j.vascn.2004.03.010 PMID: 15385089

8. Kakimoto M, Kawaguchi M, Sakamoto T, Inoue S, Furuya H, Nakamura M, et al. Evaluation of rapid ischemic preconditioning in a rabbit model of spinal cord ischemia. Anesthesiology. 2003;99:1112-1117. https://doi.org/10.1097/00000542-200311000-00017 PMID: 14576547

9. Mazensky D, Radonak J, Danko J, Petrovova E, Frankovicova M. Anatomical study of blood supply to the spinal cord in the rabbit. Spinal Cord. 2011;49:525-528. https://doi.org/10.1038/sc.2010.161 PMID: 21079621

10. Mazensky D, Flesarova S, Sulla I. Arterial Blood Supply to the Spinal Cord in Animal Models of Spinal Cord Injury. A Review. Anat Rec (Hoboken). 2017;300:2091-2106. https://doi.org/10.1002/ar.23694 PMID: 28972696

11. Mazensky D, Danko J, Petrovova E, Mechirova E, Prokes M. Arterial peculiarities of the thoracolumbar spinal cord in rabbit. Anat Histol Embryol. 2014;43:346-351. https://doi.org/10.1111/ahe.12081 PMID: 23952724

12. Liu W, Duan CZ, He XY. Anatomic study of arterial supply to the spinal cord in New Zealand White rabbits. Chin J Neuromed. 2016;15:685-689.

13. Fast JP, Perkins MG, Pearce RA, Mecozzi S. Fluoropolymer-based emulsions for the intravenous delivery of sevoflurane. Anesthesiology. 2008;109:651-

656. https://doi.org/10.1097/ALN.0b013e31818630ff PMID: 18813044

14. Huang H, Li R, Liu J, Zhang W, Liao T, Yi X. A phase I, dose-escalation trial evaluating the safety and efficacy of emulsified isoflurane in healthy human volunteers. Anesthesiology. 2014;120:614-625. https://doi.org/10.1097/aln.0000000000000044 PMID: 24162462

15. Yang XL, Ma HX, Yang ZB, Liu AJ, Luo NF, Zhang WS, et al. Comparison of minimum alveolar concentration between intravenous isoflurane lipid emulsion and inhaled isoflurane in dogs. Anesthesiology. 2006;104:482-487. https://doi.org/10.1097/00000542-200603000-00015 PMID: 16508395

16. Zhou JX, Luo NF, Liang XM, Liu J. The efficacy and safety of intravenous emulsified isoflurane in rats. Anesth Analg. 2006;102:129-134. https://doi.org/10.1213/01.ane.0000189612.24147.07 PMID: 16368817

17. Natalini CC, Da Silva Serpa PB, Cavalcanti RL, Polydoro AS, Griffith JE, Santos LC, et al. General anesthesia with an injectable 8% v/v sevoflurane lipid emulsion administered intravenously to dogs. Vet Anaesth Analg. 2016;43:271-280. https://doi.org/10.1111/vaa.12317 PMID: 26451867

18. Yang J, Li Z, Gong CY, Chai YF, Li T, Li GH, et al. A model for the preferential delivery of isoflurane to the spinal cord of the goat. Vet J. 2011;187:239-244. https://doi.org/10.1016/j.tvjl.2010.01.004 PMID: 20739204

19. White D. Uses of MAC. Br J Anaesth. 2003;91:167-169.

https://doi.org/10.1093/bja/aeg160 PMID: 12878612

20. Qin C, Wei H. Laboratory Animal Sciences. 2th Edition. People's Medical Publishing House; 2010.

21. Miller RD, Eriksson LI, Fleisher LA. Miller's Anesthesia. 8th Edition. Philadelphia: Elsevier Churchill Livingstone, 2014.

22. Soares JH, Brosnan RJ, Fukushima FB, Hodges J, Liu H. Solubility of haloether anesthetics in human and animal blood. Anesthesiology. 2012;117:48-55. https://doi.org/10.1097/ALN.0b013e3182557cc9 PMID: 22510863

23. Yu RG, Zhou JX, Liu J. Prediction of volatile anaesthetic solubility in blood and priming fluids for extracorporeal circulation. Br J Anaesth. 2001;86:338-344. https://doi.org/10.1093/bja/86.3.338 PMID: 11573521

24. Zhang P, Yang P, Ye L, Zhu T. Spinal Cord is the Primary Site of Action for Skeletal Muscle Relaxation by Sevoflurane. Spine (Phila Pa 1976). 2017;42:E1275-e1281. https://doi.org/10.1097/brs.0000000000002162 PMID: 28338578

Fig 1 Diagram of the differential anesthetic delivery rabbit model

Fig 2 The positive correlation between isoflurane blood/gas partition coefficient and the volume of emulsified isoflurane delivered

Fig 3 The positive correlation between sevoflurane blood/gas partition coefficient and the volume of emulsified sevoflurane delivered

Table 1 The position of catheter tips in two groups

Group	T_2	T_3	Total
Isoflurane	2 (25%)	6 (75%)	8
Sevoflurane	4 (50%)	4 (50%)	8
Total	6	10	16
P value	0.608		

Table 2 The mean atrial pressure (MAP) of femoral and central ear artery among different time points in two groups (mean ± SD)

Group	Position	After induction	Midpoint of infusion	End of the study
Isoflurane	Central ear artery	90.4±2.3	86.3±5.5	87.5±4.5
	Femoral artery	92.3±3.6	74.6±6.1$^{\#\&}$	88.4±4.4
Sevoflurane	Central ear artery	88.1±5.1	84.6±7.5	84.3±5.4
	Femoral artery	90.3±5.0	71.4±4.3$^{\#\&}$	86.1±4.8

$^{\#}$*P*<0.001, midpoint of infusion vs after induction

$^{\&}$*P*<0.001, midpoint of infusion vs end of the study

Table 3 The heart rate (HR) among different time points in two groups (mean ± SD)

Group	After induction	Midpoint of infusion	End of the study
Isoflurane	203±11	201±5	212±11
Sevoflurane	215±11	207±9	220±13

Table 4 The partial pressure of isoflurane and sevoflurane (mmHg) in the jugular and femoral vein in two groups (mean ± SD)

Group	P_j jugular vein	P_f femoral vein	Ratio1 (P_j/ P_f)%	Ratio2 (P_f/ P_j)	*P* value
Emulsified isoflurane (8mg/kg/h)	3.91±1.11	12.61±1.60	31.28±9.17%	3.48±1.13	<0.001
Emulsified sevoflurane (12mg/kg/h)	3.89±1.00	19.92±1.84	19.40±4.46%	5.34±1.18	<0.001

Clkk here to download Figure Figure 1 diag1am.jpg

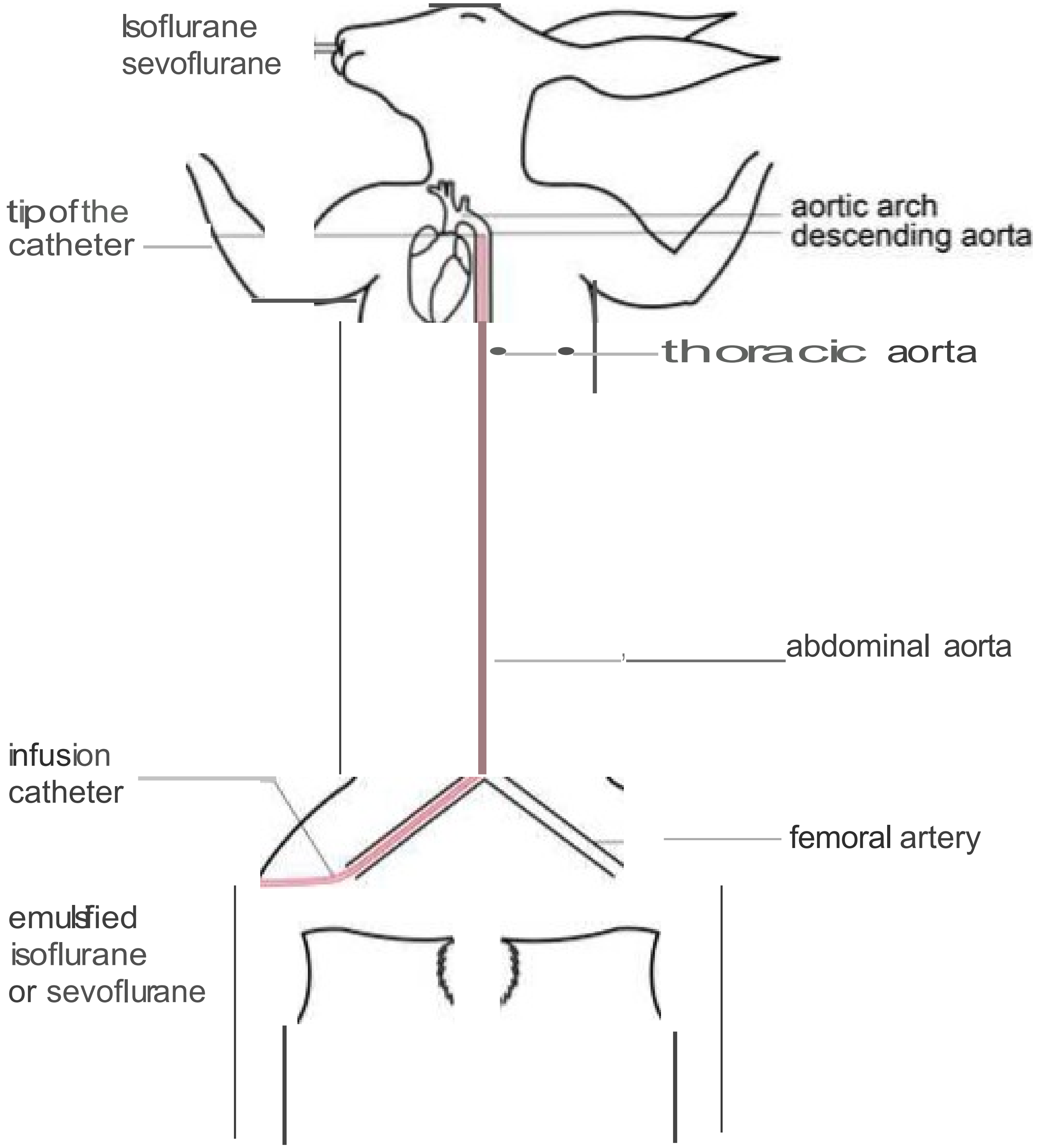

Figure 2 Click here to download Figure Figure2-iso.JPG

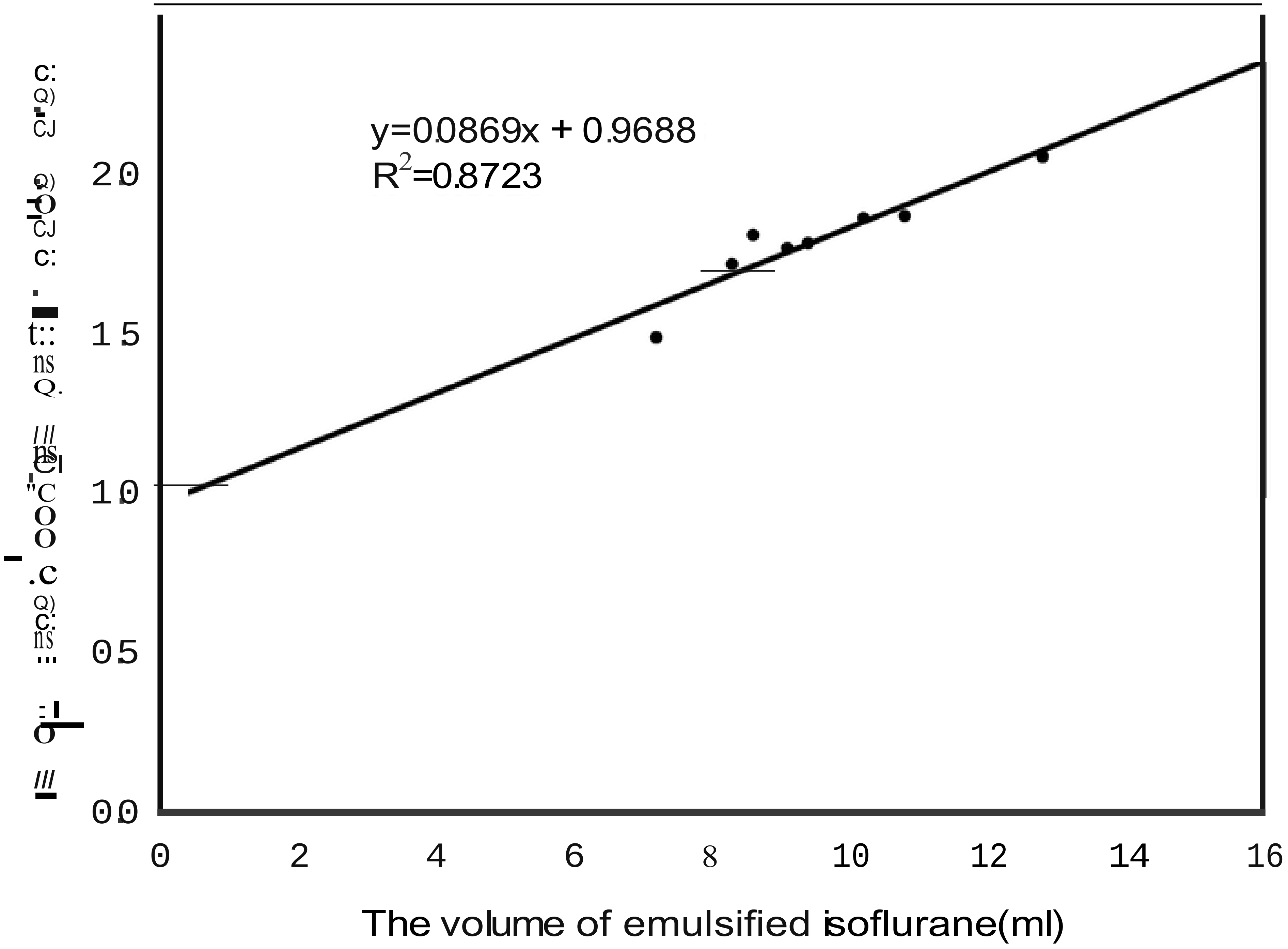

Figure 3

Click he1e to dCflYrlbd Figu1e Figu1e3-sevo.JPG

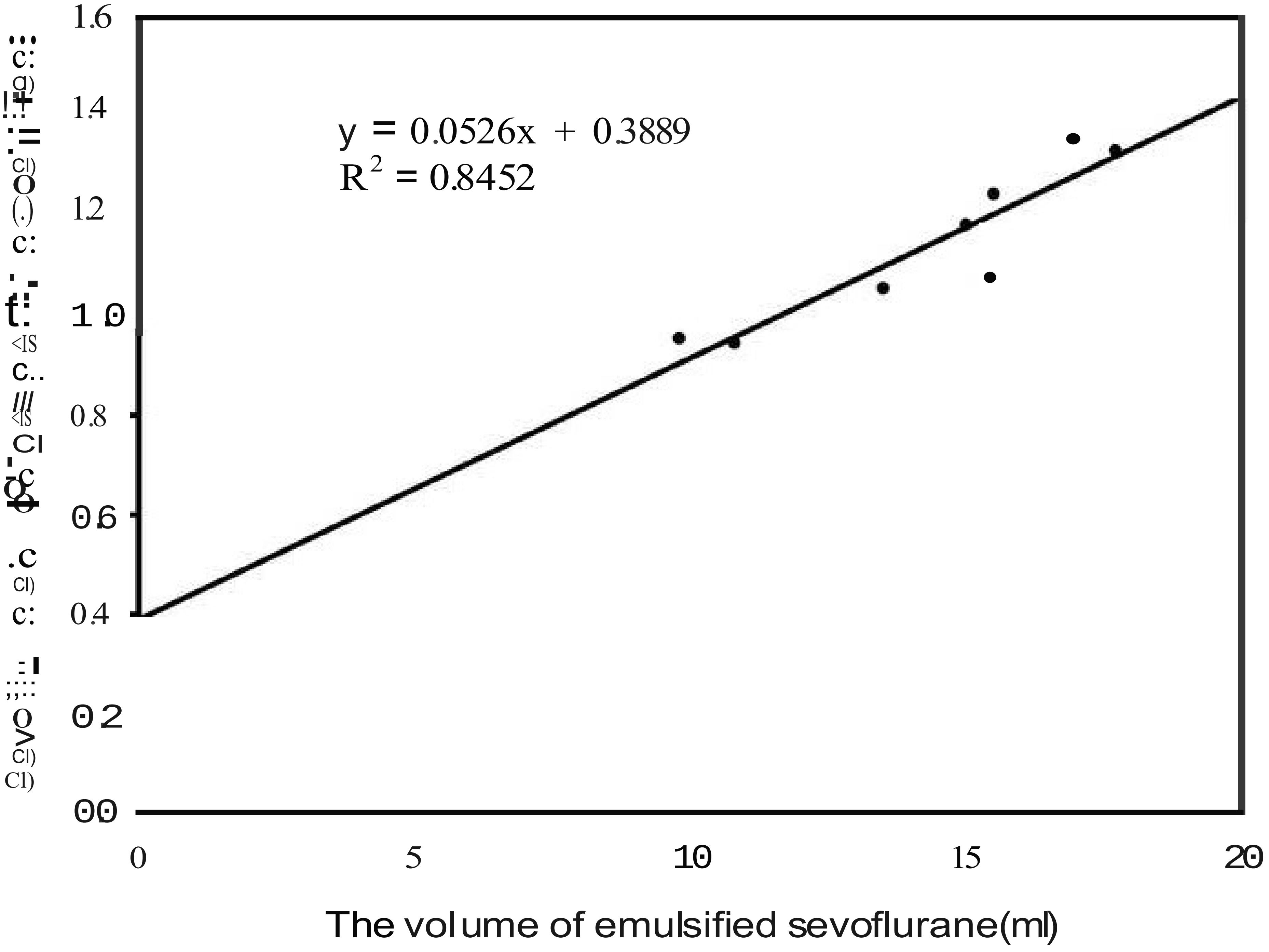

Supplement: Attachment — Submitted filename: Zhang etal_emulsified VA_rabbitt_reviewer edit.pdf [file pone.0223700.s002.pdf]
