# Supplementary material for: Development of a simple method for differential delivery of volatile anesthetics to the spinal cord of the rabbit
Source: PLoS One. 2020 Feb 24;15(2):e0223700. doi: 10.1371/journal.pone.0223700 (PMC7039460; doi:10.1371/journal.pone.0223700)
Supplement: Attachment — Submitted filename: Rebuttal letter.docx [file pone.0223700.s003.docx]

Dear Dr. Solevåg,

Authors would like to thank all reviewers for taking time to review our manuscript and for their valuable comments and constructive suggestions.

Based on the suggestions from reviewers, we have extensively revised the manuscript, edited English language, and discussed the issues associated with animal welfare. Moreover, all data underlying the findings have also been submitted as Supporting Information.

We hope that our revised manuscript now meets the high standards of the PLOS ONE. Below are our point-to-point response to reviewers’ comments.

Looking forward to hearing from you soon,

With kindest regards,

Yours Sincerely

Ting Xu, MD

Responses to the comments of Reviewer #1

The methods manuscript by Zhang et al uses a rabbit mode to differentially deliver volatile anesthetics to the brain versus spinal cord using emulsified agents infused into the descending aorta. Although this has been achieved in prior studies using brain bypass, the main advantage of this model is that it is simple and minimally invasive. However the authors statements that prior models suffered from "CNS damage" is not supported at least in prior goat models where MAC values remained normal before and following bypass, or when bypass was implemented and equal amounts of anesthetic were delivered to the brain and spinal cord. Therefore these comments should be omitted or toned down, and rather the authors should highlight the real advantage of being simpler, less labor intensive and less costly.

**Response:** We agree with the reviewer that the trauma and change of the cerebral and spinal cord circulation caused by the brain bypass don’t lead to CNS damage and MAC change. Previous goat model has shown normal isoflurane MAC before and after brain bypass [1]. Moreover, when isoflurane was administrated to both the bypassed brain and torso of the goats, MAC also did not change [1]. In our revised manuscript, we have modified the sentence according to the reviewer’s suggestions. We removed the sentences “with an intact CNS and circulatory system” and “anesthetic effect with no apparent pathological damage to the CNS and circulatory system” in the revised manuscript.

Generally, bypass models need special and expensive equipment (like membrane oxygenator and roller pump etc.), and the procedure is complex and may cause more traumatic injuries. Based on reviewer’s suggestion, we highlighted the advantage of our method being simple, minimally invasive, less labor intensive and less costly.

The reported differences in partial pressures of volatile anesthetics in the femoral vein ("spinal cord") were 3.5 to 5 fold greater than in jugular vein ("brain") which is very good separation similar to bypass models. However, this model is limited in that unlike bypass models, anesthetic cannot be regulated to the brain. THIS PRESENTS A SERIOUS ANIMAL WELFARE CONCERN, especially for VA with low solubility, like sevoflurane used in the present study. That is, with peri-MAC concentrations delivered to the spinal cord, anesthetic concentrations to the brain reach low enough concentrations to permit the rabbits to become conscious during the experiment. This welfare concern and the fact that brain anesthetic cannot be regulated is a major limitation to the model the authors failed to mention.

The minimal invasive techniques make this less concerning, however it is still concerning. Perhaps if the rabbits were acclimated and trained to lie quietly in a restrainer with the mask on before conducting the procedure, this would eliminate most of the animal welfare issues. However I cannot say if this is within a rabbits behavioural repertoire.

**Response:** First of all, we support animal welfare and followed the laboratory animal welfare regulation during the experiment. Because of our poor English, the experimental procedures were not very described clearly in our previous version of the manuscript.

We have added detailed information in the *Materials and Methods* section. (i.) Before intubation, the endotracheal tube was coated with 1% dicaine gel (long-acting local anesthetic) for airway topical anesthesia, which could increase the tolerance for endotracheal tube [2, 3]. (Line 68-70) (ii.) Local anesthetic (1% lidocaine+0.5% ropivacaine) was injected into the skin before surgical exposure. The local anesthetic wound infiltration can provide rabbit extra analgesia during the whole procedure. (Line 73-74) (iii.) Isoflurane and sevoflurane 1.0 MAC were administrated via vaporizer to keep the rabbit unconscious and immobile during the experiment. But in order to wash out the volatile anesthetics (VAs), isoflurane and sevoflurane were discontinued, 15-20min before initiating infusion of the emulsified agents (Figure 1) (Line 101-103).

Figure 1 The flowchart

We agree that our model has limitation that VAs cannot be regulated to the brain and the VAs concentration in the brain was equivalent to 0.2-0.3MAC, when emulsified VAs was preferentially diffused to the spinal cord (Figure 1). The lower VAs concentration in the brain was only enough to provide hypnosis or sedation [4], which might bring potential animal welfare concerns. We really thank reviewer’s constructive comments, and there are three reasons to eliminate most of the animal welfare issues.

As the reviewer mentioned, rabbits have natural tolerance for restraint and they naturally stay immobile in a confined space [5]. In our study, instead of fixing on the bench, all rabbits were unlocked and wrapped up by a surgical towel with slightly manual restrain (Figure 2) during wash-out and infusion periods, which not only help to relieve pain and alleviate stress response, but also make them immobility. Taking advantage of the rabbit’s special behavior, conscious rabbit can finish cerebral MRI and abdominal ultrasound [5, 6]. On the other hand, airway topical anesthesia and local anesthetic wound infiltration were used to provide extra analgesia and enhance the rabbit’s tolerance for pain stimulus. Local anesthesia can be considered to be a positive aspect of animal welfare in our study. Lastly, except wash-out and infusion periods, all rabbits were under isoflurane or sevoflurane (about 1MAC) general anesthesia administrated via vaporizer in most of the procedure (Figure 1).

We also have discussed the limitation and animal welfare concerns in our revised manuscript (Line 208-223).

Figure 2 The rabbit was wrapped up by a surgical towel

Last, while I am sympathetic to the language barriers non-english speaking groups face, the manuscript should be reviewed and edited by a fluent english writer BEFORE submission. While some parts are written fairly well, I still extensively edited the manuscript (see attached file) for basic syntax, grammar and clarity. It is not appropriate to place this burden on a scientific reviewer, and moreover the authors should have a vested interest in submitting a comprehensible manuscript to maximize chances of being accepted.

**Response:** We thank the reviewer again for editing the English language, which is of great help to improve the quality of our manuscript. Before submission, our revised manuscript has been reviewed and proofread by a fluent English writer.

We also have responded, point by point to the comments marked on the original manuscript as blow:

[but this model cannot distinguish between spinal cord and peripheral nerve effects]

**Response:** The phrase “peripheral nerves” is omitted.

[8 rabbits per group?]

**Response:** The sentence “Sixteen New Zealand White rabbits (male and female) weighting 2.0-3.0kg were randomly assigned to the isoflurane and or sevoflurane group (8 each).” is added in Line 64-66.

[in which cases/for what was each of these two test used?].

**Response:** We add the sentence “Other continuous variable data (the weight of the rabbit, the length of the catheter and the partial pressure of isoflurane and sevoflurane) were analyzed by student’s t test. χ2 test was used to evaluate the position of catheter tips.” in Line 120-122.

[this sentence is unclear please clarify]

**Response:** The modified sentence is “Based on “volume fraction partition coefficient” theory, when 1ml of lipid emulsion was administrated to those three animals, the increase of rabbit λb/g of isoflurane might be larger than that of goat or dog”(Line 204-207).

[but it is already known VA act primarily in the spinal cord to exert immobility, so how would this add to our body of knowledge?]

**Response:** We agree that VA act primarily in the spinal cord to produce immobility [1, 7]. We accept the reviewer’s suggestions and omit the sentence “This allows us to determine if an anaesthesia action is due to brain or spinal cord effects”.

Since the unique homosegmental blood supply to the spinal cord in rabbit, different spinal segments (for example, thoracic, lumbar or sacro-coccygeal spinal cord) can be selectively anesthetized through adjusting the placement of the infusion catheter. Thus, this model may be used to investigate the effects of VA on different spinal segments.

Reference

1.	Antognini JF, Schwartz K. Exaggerated anesthetic requirements in the preferentially anesthetized brain. Anesthesiology. 1993;79:1244-1249. <https://doi.org/00000542-199312000-00015> PMID: 8267200

2.	Chung DC, Mainland PA, Kong AS. Anesthesia of the airway by aspiration of lidocaine. Can J Anaesth. 1999;46:215-219. <https://doi.org/10.1007/bf03012598> PMID: 10210043

3.	Yang J, Li Z, Gong CY, Chai YF, Li T, Li GH, et al. A model for the preferential delivery of isoflurane to the spinal cord of the goat. Vet J. 2011;187:239-244. <https://doi.org/10.1016/j.tvjl.2010.01.004> PMID: 20739204

4.	Miller RD, Eriksson LI, Fleisher LA. Miller's Anesthesia 8th. 2010;17章:308. <https://doi.org/10.1016/j.jss.2011.10.001> PMID: 22225976

5.	Weiss C, Procissi D, Power JM, Disterhoft JF. The rabbit as a behavioral model system for magnetic resonance imaging. J Neurosci Methods. 2018;300:196-205. <https://doi.org/10.1016/j.jneumeth.2017.05.021> PMID: 28552515

6.	Schroeder MP, Weiss C, Procissi D, Wang L, Disterhoft JF. Activity-induced manganese-dependent MRI (AIM-MRI) and functional MRI in awake rabbits during somatosensory stimulation. Neuroimage. 2016;126:72-80. <https://doi.org/10.1016/j.neuroimage.2015.11.033> PMID: 26589332

7.	Yang J, Chai YF, Gong CY, Li GH, Luo N, Luo NF, et al. Further proof that the spinal cord, and not the brain, mediates the immobility produced by inhaled anesthetics. Anesthesiology. 2009;110:591-595. <https://doi.org/10.1097/ALN.0b013e3181974bfd> PMID: 19212265
