# Supplementary material for: Development of a simple method for differential delivery of volatile anesthetics to the spinal cord of the rabbit
Source: PLoS One. 2020 Feb 24;15(2):e0223700. doi: 10.1371/journal.pone.0223700 (PMC7039460; doi:10.1371/journal.pone.0223700)
Supplement: Attachment — Submitted filename: Rebuttal letter revised 2.docx [file pone.0223700.s004.docx]

PONE-D-19-27037

Development of a simple method for differential delivery of volatile anesthetics to the spinal cord of the rabbit

PLOS ONE

Dear Dr. Solevåg,

We once again thank the reviewers for their insightful review and valuable comments.

According to the suggestions received, we have added three references in the *Discussion* section (reference 25, 26, 28). The sentence “All rabbits stayed immobile and did not display any signs of distress or discomfort during the procedure” was also added (lines 225-227) to support our study.

Furthermore, we have had our revised manuscript edited by a native English-speaking editor to improve the language quality.

We hope that our revised manuscript now meets the high standards of PLOS ONE.

We look forward to hearing from you soon.

Sincerely

Ting Xu, MD

Reviewer #1: Overall the authors have addressed comments and concerns, with some minor ones remaining although I feel these do not require re-review:

The manuscript still needs some minor English language editing including the revised text, however it is much improved.

The authors make good points about animal welfare concerns however the comments are mostly speculative without reference to what they actually observed at the relevant time points. Please add something to the effect "animals did not display signs of distress or discomfort during the infusion and post-infusion time periods" (assuming this was true).

**Response:** We agree with the reviewer and have performed some modifications in our revised manuscript. Three references (reference 25, 26, 28) was added in the *Discussion* section to support our results. In addition, the sentence “All rabbits stayed immobile and did not display any signs of distress or discomfort during the procedure” was also added (lines 225-227).
